# Learning a covert sequence of effector movements: limits to its acquisition

**DOI:** 10.1007/s00426-023-01855-3

**Published:** 2023-07-09

**Authors:** Leif Johannsen, Iring Koch

**Affiliations:** 1 Cognitive and Experimental Psychology, Institute of Psychology, RWTH Aachen University, Jägerstrasse 17/19, 52066 Aachen, Germany; 2 Department of Psychology, Durham University, Durham, UK

## Abstract

Sequence learning in serial reaction time (SRT) tasks is an established, lab-based experimental paradigm to study acquisition and transfer of skills based on the detection of predictable regularities in stimulus and motor response sequences. Participants learn a sequence of targets and responses to these targets by associating the responses with subsequently presented targets. In the traditional paradigm, however, actions and response targets are directly related. In contrast, the present study asked whether participants would demonstrate acquisition of a sequence of effector movements of the left vs. right hand (e.g., hand sequence learning), whilst the actual targets and associated finger responses are unpredictable. Twenty-seven young adults performed a SRT task to visually presented characters with the index or middle fingers of both hands. While the specific fingers to respond with were randomly selected for each target presentation, both hands followed a covert sequence. We asked whether participants would learn the underlying hand sequence as demonstrated by shortened response latencies and increased accuracy compared to a fully randomized hand sequence. The results show sequence-specific learning effects. However, categorization of hand responses depending on the previous response suggested that learning occurred predominantly for subsequent finger responses of the same hand, which added to general hand-based priming. Nevertheless, a marginally significant effect was observed even for predictable shifts between hands when homologous fingers were involved. Our results thus suggest that humans are able to benefit from predictable within-hand finger shifts but less so for predicted shifts between hands.

## Introduction

Learning to produce a series of responses in a specific order to achieve a desired goal is a fundamental capability of humans, which enables performing complex movements and adapting to new environments and situations. The serial-reaction-time (SRT) task (Nissen & Bullemer, 1987) has been well established as a lab-based paradigm to investigate the formation of procedural and implicit knowledge during the acquisition of a sensorimotor skill. In the SRT paradigm, individual manual key presses are required in response to an initially apparently random sequence of visual or auditory stimuli, for example when four adjacent reaction buttons are spatially assigned to four stimulus locations or identities. Experience with seemingly irregular but actually predictable stimulus sequences across several blocks decreases response latency and may often result in conscious awareness of the sequence compared to randomized, unpredictable series of stimulus sequences. This demonstrates improved prediction of stimulus characteristics, such as location and other features, but also preparation of a response to any upcoming stimuli (Curran & Keele, 1993; Nissen & Bullemer, 1987). When learning has progressed, performance reductions (i.e. due to slower response latencies and/or increased percent error) in blocks with unpredictable stimuli are interpreted as “negative transfer” (see, e.g., Abrahamse et al., 2010; Dienes & Berry, 1997; Keele et al., 2003; Schwarb & Schumacher, 2012 for reviews) in the sense that any previously acquired procedural knowledge cannot be used for the prediction of subsequent stimuli. Note, however, that conscious awareness of the hidden regularities in a sequence of target stimuli during an SRT task might not be necessary for the learning to occur and may be achieved only partially or not at all in the majority of participants (Dienes & Berry, 1997; Esser et al., 2022; Keele et al., 2003; Shanks & St. John, 1994, for reviews).

During sequence learning, integrated stimulus-, and response-related information is the basis of the acquired performance improvements. Willingham et al. (2000) observed that for sequence learning the series of response locations may be more relevant than the series of finger movements (see also Koch & Hoffmann, 2000). It seems, however, that both the sequence of actions as well as the sequence of response locations may be acquired in parallel (Witt & Willingham, 2006). A distinction has been made between types of effector-dependent sequence representations and effector-independent sequence representations (Andresen & Marsolek, 2012; Berner & Hoffman, 2009). For example, Verwey and Wright (2004) reported evidence for both an effector-dependent as well as an effector-independent sequence learning component. Subsequently, Verwey and Clegg (2005) demonstrated, however, that effector-dependent sequence learning occurs only when the fingers of one hand are actually moved during learning. Therefore, they proposed that effector-dependent sequence learning rests on the mechanical interactions between the fingers of one hand. Similarly, Berner and Hoffman (2009) demonstrated that after extensive practice, in which participants trained not only an overall sequence but also sub-sequences for each hand in the sense that the fingers in each hand were exposed to an individual sequence, effector-specific sequence knowledge can be acquired in a SRT task.

A relevant question refers to the extent of the action representations involved in successful predictions when performing. For example, how effector-specific is the learning effect? A hierarchical account of sequence control might assume that other components of the body, such as the hand to which the fingers as effectors belong to, are also represented and that an alternating sequence of finger actions can be generalized to action sequences of the hand, the arm, or even the side of the body. Transfer of sequence knowledge between effectors has been reported (Park & Shea, 2002) and shows that the acquired knowledge is not limited to just the end-effector (Kovacs et al., 2009). In the typical transfer studies, however, the association between the features of the response targets, such as location, identity or other characteristics, and the respective effectors was direct and overt. In contrast, it is unknown if the cognitive system can acquire an association between effectors and targets that is covert in the sense that overt responses are random, while the limbs to which the effectors belong to follow a covert sequence.

To our knowledge a similar approach has not been realized up to now. Examining the learning of a hand sequence in the absence of a specific finger sequence would represent a type of hand-specific learning independent from the overt individual responses. There are previous demonstrations of learning of more abstract sequences, for example sequences of stimulus categories (e.g. Goschke & Bolte, 2012) and sequences of tasks (e.g. Koch et al., 2006), but these targeted aspects of attentional stimulus selection, such as the focus of attention to relevant stimuli in the environment. These studies did not consider patterns of motor preparation relating to the hands, such as the activation and coordination of neural circuits and muscle groups.

In choice reaction time tasks, sequential effects caused by the repetition or alternation of stimuli and/or responses can be found in response latencies (Kirby, 1976). These sequential effects have been associated with mechanisms of learning that might be involved in sequence learning too (Jones et al., 2013; Lee et al., 2016; Soetens et al., 2004), for example automatic hand-based response priming when a response of the fingers of the same hand is repeated (Adam & Koch, 2009). The task in the present study can be considered to resemble a hand-based two-alternative forced-choice task, despite the four fingers involved in the overt responses, where the processes driving repetition and alternation effects might act in analogy to finger choice reaction time tasks. Therefore, repetitions or alternations between the hands during practicing might result in distinct effects on performance. For example, Trapp et al. (2012) investigated the costs of alternating the hands in a four finger, bimanual sequence learning task and reported costs of shifting hands in terms of increased response latencies when sequential key presses of homologous fingers had to be performed. The authors’ explanation for the occurrence of the costs of hand alternations assumed mutual inhibition between the motor centres of the two hemispheres as a possible cause (Trapp et al., 2012). Trapp et al. (2012) focussed, however, on shifts between homologous fingers only and did not consider non-homologous finger pairings, the contrast of which will be considered in the present study. A finger response to a specific target requires inhibition of any unwanted, incorrect finger responses. Macdonald et al. (2012) showed that inhibitory coupling between homologous pairings is stronger compared to couplings between non-homologous finger pairings. Thus, in situations, where sequential responses are required, the necessity to overcome stronger inhibitory coupling and subsequent instantiation of a homologous finger response may result in increased performance costs for these pairings when hands are alternated. In a complementary fashion, the cost of alternating hands could be lower when non-homologous fingers need to be activated in sequence. Perez et al. (2007) reported that learning in a SRT task reduces interhemispheric inhibition, which could facilitate transfer of learning between hands (Paparella et al., 2023). These observations may imply that the greater the amount of learning and transfer in a SRT task, the greater a resulting reduction in interhemispheric inhibition. With reduced interhemispheric inhibition, excitatory interhemispheric mechanisms could grow in influence (Bloom & Hynd, 2005) and facilitate predictive interlimb integration in the learning of effector sequences.

The present study follows an approach that is similar in spirit to that of a recent study by Koch et al. (2020), where the authors examined the learning of predictable switches between perceptual modalities while performing random manual responses to either visual or auditory targets. Against their expectations, however, they did not find evidence for a performance benefit of modality-specific sequence learning with random manual responses (Koch et al., 2020). In the present study, we expected that participants would acquire knowledge of a covert, predictable sequence of shifts between the two hands while the responses of the fingers as end-effectors were (largely) unpredictable. In concrete terms, performance should be improved, both in terms of shorter response latencies and lower percent error, during a predictable hand sequence compared to a random sequence of hand shifts. Further, based on the reported effects of hand alternations in sequential response tasks, we surmised that the type of hand shifts in terms of the specific fingers involved would moderate any learning effects. In concrete terms, we expected that hand repetitions would be easier to predict and therefore should have a greater effect compared to that when predicting hand shifts. With respect to hand shifts with homologous and non-homologous finger pairings, we expected greater learning effects in non-homologous hand shifts due to initially lower levels of interhemispheric inhibition.

## Methods

### Participants

The age inclusion criterion was an age between 18 and 27 years to recruit younger adults only. We recruited 27 participants (22 females and 5 males; 23 right-handed, 4 left-handed; average age = 22.4 ± 2.7 years) from the RWTH Aachen University. The research ethics review board of the medical faculty granted ethical approval for this study (EK 322/19). All participants provided written informed consent before inclusion in the study according to the Declaration of Helsinki.

### Stimuli and task

The administration of the experimental stimuli was programmed in MATLAB using the Psychophysics toolbox (Kleiner et al., 2007). The target stimuli were the lowercase characters “a”, “d”, “j”, and “l”, which were mapped from left to right to the corresponding keys on a German QWERTZ-keyboard. The left middle finger was placed on the “a”, the left index finger on the “d”, right index finger on the “j”, and the right middle finger on the “l”. Fingers had to remain on the designated keys during the entire duration of a block. All single respective response targets were presented at the same location, that is at the centre of a computer screen, until a response was made. Following a response after an inter-trial interval of 200 ms, the next stimulus was presented. If an incorrect response was made, a “Falsch!” (German for “Incorrect!”) was presented as error feedback for an additional 300 ms. Participants were instructed to press the correct key as fast as possible following stimulus presentation and to release the key afterwards. The accuracy of a response was also emphasized.

Instead of arranging all four target stimuli (and corresponding fingers) into a sequence 12 responses, only a 12-element sequence of alternations between the two response hands was predetermined according to these patterns: *right*-*right*-left-left-*right*-left-*right*-left-*right*-*right*-left-left (for right-handed participants) or *left*-*left*-right-right-*left*-right-*left*-right-*left*-*left*-right-right (for left-handed participants). For left-handers, a mirrored hand sequence was applied to enable interpretation of the sequences in terms of hand dominance. In this pattern, eight shifts between the two hands occurred and 4 repetitions of the same hand. When a specific hand was called, each of the two fingers of that hand had a 50% chance to be drawn at random. Which of the two fingers occurred was randomly determined online, so that the exact sequence of finger actions varied individually for each participant. The only constraint was that if a hand was repeated then the other finger of that hand was committed as a subsequent response. In other words, the same response could not occur in immediate repetition. This constraint, which adheres to a similar constraint employed in previous sequence learning experiments (Blotenberg et al., 2018; Zirngibl & Koch, 2002), was valid also in those control blocks, in which a random hand sequence was generated. However, it also meant that in hand repetitions, if one finger was used to respond, then the other finger of the same hand was fully predictable to occur next. Randomized sequences were not subject to any additional constraints except for the one that also applied to regular sequences.

Each 12-element sequence was cyclically repeated 4 times resulting in a predetermined sequence of 48 individual responses per mini-block, and one block comprised 5 mini-blocks, so that for a single block 240 (= 12*4*5) individual responses were requested. The experiment paused after each mini-block and every block but participants were instructed to take an actual break after blocks only, if required. In total 12 blocks were assessed. While the hand sequence followed a fixed pattern of the respective assigned sequence in the Blocks 3 to 9 and Blocks 11 and 12, randomized hand sequences were presented in Blocks 1, 2 and 10. For these three blocks, each mini-block consisted of newly randomized sequences, so that every participant received a unique set of three randomized blocks. Participants performed a practice block of 32 random stimuli in 4 mini-blocks of 8 responses at the start of the experiment to familiarize themselves with the experimental procedure. After data collection had been completed, a structured follow-up interview was conducted with each participant to ask whether they had detected any regularities on the sequence of target stimuli or whether they could even recall parts of or their entire sequence explicitly.

### Design and outcome parameters

Predictability of the hand sequence (random vs. predictable) was the independent within-subject variable. Participants’ percentage error and the response latency of a key press following target stimulus presentation were extracted to determine any hand sequence-specific learning effects within participants. The increase in response times and error rates from Blocks 8 and 9 to Block 10 and the decrease from Block 10 to Blocks 11 and 12 was interpreted as a measure of how well the respective hand sequence was internalized. Thus, learning of the hand sequence was determined as the difference between the average of Blocks 8, 9, 11, and 12 and performance in the random Block 10.

### Statistical analysis

For the calculation of the average response latencies, the first trial of each block, all trials with incorrect responses and, to avoid the influence of post-error slowing on latencies, all correct responses following an error trial were excluded from analysis. All statistical computations were performed in R Studio 1.1.456. Paired t-tests were applied to test for a significant change in the observed learning effects against a hypothetical baseline effect of 0. An alpha level of 5% was used to determine statistical significance.

Performance was evaluated both in terms of hand shifts and repetitions and in terms of the specific shifts and repetitions between single fingers. Each response was sorted into one of three categories depending on whether a response followed a response of the same or the other hand and, in case of the other hand, whether it was the homologous finger or not: different finger same hand, different finger different hand, same finger different hand (note that condition “same finger in same hand” could not occur). For the learning effects of both primary outcome parameters, type of shift was included in separate ANOVAs as a 3-level within-subject factor. Bonferroni-adjusted single comparisons were performed across shift types, where necessary.

## Results

None of the 27 participants reported any subjective impression that some regularities in the sequence of the hands occurred. Six participants mentioned that regularities in the sequence of response targets had been present but they could not name them. An additional seven participants reported patterns of a possible target combination such as one or more of the quadruples “ljda” (3x), “jlda” (3x), “jlad” (1x), “adjl” (1x), or “aldj” (1x).

**Fig. 1:**
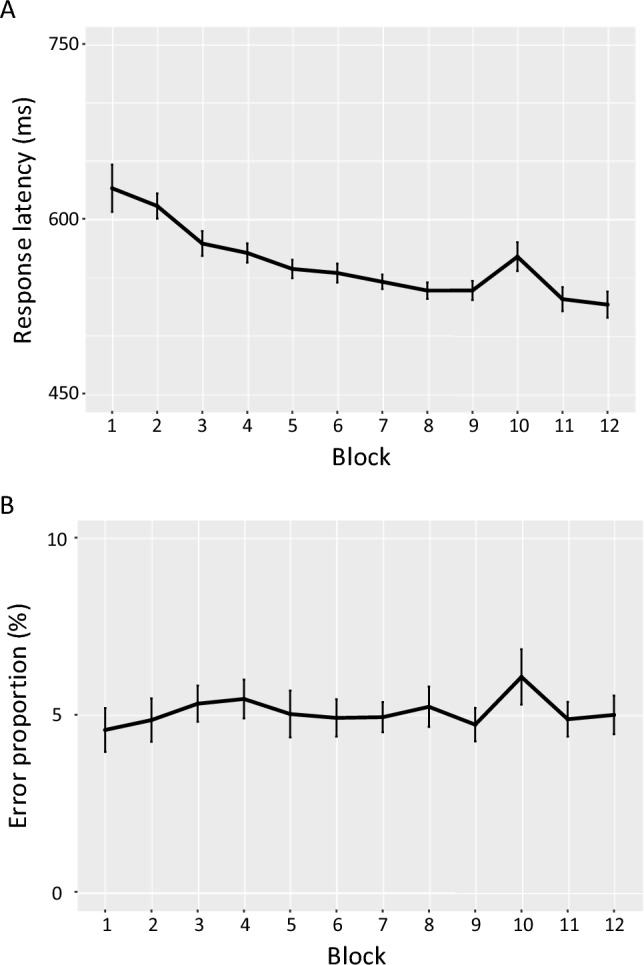
Performance curves of the response latencies (**A**) and percent error (**B**). In Blocks 1, 2 and 10, the sequence of stimuli was random, while in the remaining Blocks 3 to 9 as well 11 and 12 the respective hand sequence was presented 20 times. Error bars represent 95% confidence intervals

In blocks with regular hand sequences, hand shifts occurred in 66.0% of trials, while hand repetitions in 34.0%. In randomized blocks, similar proportions occurred: hand shifts (67.3%), hand repetitions (32.7%). The response latency showed a strong sequence-specific learning effect of 34 ms (SD 30; Blocks 8, 9, 11, and 12: mean = 533 ms, SD 68; Block 10: 567 ms, SD 65; *t*(26) = 5.89, *p* < 0.001, dz = 1.13). A learning effect of 1.12% (SD 2.07) was also found for the percent errors (PE; Blocks 8, 9, 11, and 12: mean = 4.95%, SD 2.18; Block 10: mean = 6.07%, SD 3.31; *t*(26) = 2.79, *p* = 0.01, dz = 0.54). Figure 1 shows the average performance curves for response latency and percent error of all participants across the 12 blocks.

**Fig. 2:**
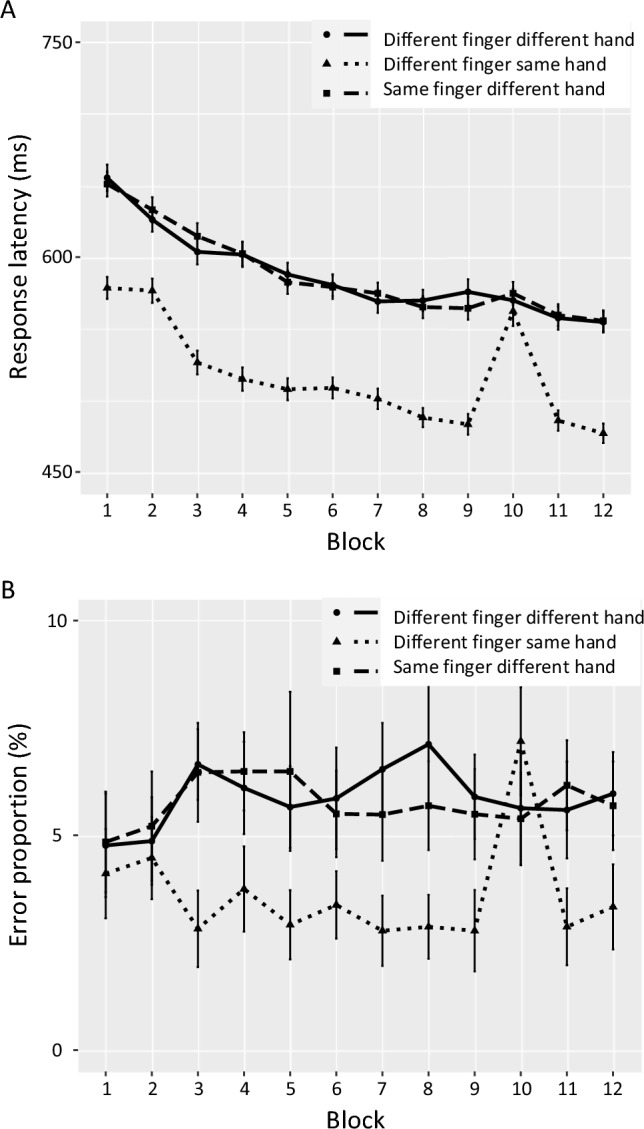
Performance curves of the response latencies (**A**) and percent error (**B**) as a function of the finger shift within or between hands. In the Blocks 1, 2 and 10, the sequence of stimuli was random, while in the remaining Blocks 3 to 9 as well 11 and 12 the respective hand sequence was presented 20 times. Error bars represent 95% confidence intervals

In blocks with regular hand sequences, finger shifts between different fingers in the same hand occurred in 34.0% of trials, shifts between same fingers of different hands in 32.8%, and shifts between different fingers in different hands in 33.2%. In randomized blocks, these proportions were comparable to sequence blocks: different fingers in the same hand (32.7%), same fingers of different hands (33.4%), and different fingers of different hands (33.9%). The type of shift influenced the learning effect in terms for percent error (*F*(2,52) = 24.00, *p* < 0.001, *ƞ*_p_^2^ = 0.48) and response latency (*F*(2,52) = 48.09, *p* < 0.001, *ƞ*_p_^2^ = 0.65). Figure 2 shows performance curves for the response latencies and percent error as a function of the finger shift. Finger shifts based on hand repetitions (different finger same hand) showed significant learning effect of hand sequence (*t*(26) = 8.77, *p* < 0.001, dz = 1.69), shifts between hands involving homologous fingers showed a tendency (*t*(26) = 1.93, *p* = 0.06, dz = 0.37), while shifts between non-homologous fingers showed no learning effect (*t*(26) = 0.99, *p* = 0.33, dz = 0.19). Only finger shifts with hand repetitions showed a learning effect for percent error (*t*(26) = 6.47, *p* < 0.001, dz = 1.25).

The response latency learning effect for shifts when one finger followed the other of the same hand was significantly greater compared to the other two types of between-hand finger shifts (contrast against different finger different hand: *t*(26) = 7.45, *p* < 0.001, dz = 1.89; contrast against same (i.e. homologous) finger different hand: *t*(26) = 8.13, *p* < 0.001, dz = 1.52). Learning effects did not differ between the two finger shifts involving a different hand (*t*(26) = 1.35, *p* = 0.56, dz = 0.25). In order to assess a potential learning effect in those two conditions involving hand shifts irrespective of whether a homologous finger was involved or not, we averaged response latency across the two types of finger shifts comprising different hands and compared the average of the Blocks 8, 9, 11, and 12 against the random Block 10. Responses latency tended to be faster in the blocks with predictable hand shifts (mean = 561 ms, SD 77) compared to unpredictable hand shifts (mean = 571 ms, SD 69), but this 10 ms learning effect was just not significant (*t*(26) = 1.73, *p* = 0.09, dz = 0.14). Yet, as we clearly predicted benefits (rather than costs) of learning to occur and therefore our hypothesis was actually directed, this effect could justifiably be tested one-tailed and thus considered significant conventionally at the alpha = 0.05 level, even though we remain cautious about a stronger interpretation.

The learning effect for percent error was significantly greater for different finger same hand shifts compared to the other two types (contrast against different finger different hand: *t*(26) = 6.26, *p* < 0.001, dz = 1.41; contrast against same finger different hand: *t*(26) = 5.63, *p* < 0.001, dz = 1.53). The other two finger shift types did not differ significantly (*t*(26) = 0.18, *p* = 1.0, dz = 0.05). A comparison of percent error between predictable hand shifts irrespective of the finger shifts involved (mean = 5.95%, SD 2.64) against unpredictable hand shifts showed no significant learning effect (mean = 5.51%, SD 3.49; *t*(26) = 1.03, *p* = 0.31, dz = 0.14). Table 1 summarizes the descriptive statistics and statistical results for the paired *t*-tests of learning effects as function of the finger shift.

**Table 1:** Descriptive statistics and sequence-specific learning effects as a function of the finger shift

	Mean Blocks 8, 9, 11 and 12 (SD)	Mean Block 10 (SD)	Learning effect (AV, SD)	Learning effect statistical comparisons
	(*n* = 27)	(*n* = 27)	(*n* = 27)	All vs. 0 (df = 26)
Shift type: different finger, different hand				
PE (%)	6.14 (2.96)	5.63 (4.16)	− 0.51 (3.34)	*t* = − 0.80, *p* = 0.43, dz = 0.15
T-TARGET (ms)	562 (79)	568 (77)	6 (29)	*t* = 0.99, *p* = 0.33, dz = 0.19
Shift type: same finger, different hand				
PE (%)	5.76 (2.72)	5.39 (3.94)	− 0.37 (2.56)	*t* = − 0.76, *p* = 0.46, dz = 0.15
T-TARGET (ms)	559 (76)	573 (65)	14 (38)	*t* = 1.93, *p* = 0.06, dz = 0.37
Shift type: different finger, same hand				
PE (%)	2.96 (1.82)	7.19 (4.15)	4.23 (3.39)	*t* = 6.47, *p* < 0.001, dz = 1.25
T-TARGET (ms)	482 (60)	562 (69)	80 (47)	*t* = 8.77, *p* < 0.001, dz = 1.69

*PE* percent error

In order to assess whether a general hand-based priming acted in the blocks with random hand sequence, we analysed the response latencies and percent error in the random Blocks 1, 2, and 10 as a function of the finger shift. Response latency was affected by the type of finger shift (*F*(2,52) = 41.12, *p* < 0.001, *ƞ*_p_^2^ = 0.61). Post-hoc single comparisons resulted in faster response latencies for finger shifts of hand repetitions (mean = 572 ms, SD 56) compared to homologous finger shifts between hands (mean = 620 ms, SD 63; *t*(26) = 7.00, *p* < 0.001, dz = 0.80) and non-homologous finger shifts between hands (mean = 614 ms, SD 66; *t*(26) = 6.97, *p* < 0.001, dz = 0.69). The other two types of finger shifts were not significantly different in their response latencies (*t*(26) = 1.25, *p* = 0.22, dz = 0.08). Finally, percent error was not affected by type of finger shift (*F*(2,52) = 0.07, *p* = 0.93, *ƞ*_p_^2^ = 0.003).

## Discussion

Using a SRT task, we exposed young adult participants to a predictable sequence of hand shifts while they had to respond to alphabetical stimuli, which were initially unpredictable. We expected that participants would improve their performance in terms of reduced response latency and reduced percent error. Both outcome parameters demonstrated clear learning effects when comparing final performance in the predictable against the unpredictable series. However, predicting shifts between hands was not universally beneficial but the benefit was largely limited to finger actions that repeated the same hand despite the fact that hand shifts occurred twice as frequently in the sequence compared to repetitions. When a regular shift between hands occurred, irrespective of whether a homologous finger to the previous one was activated or not, finger responses showed the tendency of a small performance advantage in terms of response latency compared to a random series of hand shifts. Against our expectations, however, hand shifts with non-homologous finger pairings did not demonstrate a greater learning effect than shifts with homologous finger pairings. Nevertheless, we replicated a general benefit of hand repetitions against both types of hand alternation.

To our knowledge, the present study may be the first to demonstrate the possibility of hand sequence learning in a SRT task, in which the hands took part of the required actions only indirectly. Learning to benefit from predictable hand sequences when only the fingers are in the focus of action suggests some abstraction from the sequence of individual stimuli and responses. Compared, for example, to predictable stimulus modality (visual vs. auditory) sequences in the context of an otherwise unpredictable manual response sequence, where no clear performance benefits could be detected (Koch et al., 2020), the present hand sequence learning paradigm may have uncovered learning at the level of the within-hand finger sequence.

It should be noted though that the constraints imposed on the stimulus sequence, disallowing immediate response repetitions, introduces some partial predictability of the response in the context of a hand repetition. That is, if a hand repetition could be predicted, it could also be inferred, after sufficient exposure to the sequence, that the next stimulus would be a different stimulus, thus calling for the other finger response on the same hand. Note that such specific learning processes could take place even in the random sequences, that is, participants could learn that the same response will not be called for in the subsequent trial. However, predicting a hand repetition in the predictable sequences, relative to unpredictable hand repetitions in the random sequence block, allowed reducing the response alternatives from three to a single option, which could be prepared during the response-stimulus interval. In contrast, in the random blocks, it could only be predicted that the same response is not possible and thus a hand shift is more likely to occur next. In contrast, when being able to predict a hand shift, this would allow reducing the number of possible responses in the next trial only from three to two. It is interesting that this partial response predictability still yielded small performance benefits (but just not significant, *p* = 0.06 for the 14 ms learning effect for shifts between homologous fingers of different hands), suggesting that some small amount of learning may actually have taken place, which might become significant with more statistical power (e.g., a larger sample size). Hence, while we currently can only speculate about the benefit of predicting a hand shift, we can clearly state that the opportunity to predict not only the hand but also, in the case of a hand repetition, the specific response, gives a substantial performance gain.

The present SRT task represents a paradigm in which the hand sequence can be acquired in an incidental learning situation, that is, without explicit instruction to use the predictable hand sequence for response preparation. It is important to note that the hand sequence included both switches and repetitions, that means that if specific benefits for repetitions were observed, then it is nevertheless consistent with a hand sequence-specific benefit. In a typical SRT task, a sequence is always without immediate response repetitions, while attention is directed at the effectors and their responding in sequence. In the present study, this aspect was kept similar as individual fingers did not repeat. In contrast, however, incidental learning was largely conditional to hand repetitions. Only when participants had learnt that the next response would involve the same hand and that finger repetitions were not possible, then the following finger response could be predicted. Thus, prediction of the hand repetition is necessary to allow subsequent finger prediction. In contrast, hypothetical prediction of a hand switch did not allow finger prediction, which indicates that that knowledge cannot be applied as easily in order to achieve a performance benefit. It is thus interesting to relate the present learning effects to those performance benefits that can be observed in an explicit response preparation paradigm using the so-called finger-cuing task (e.g., Adam & Koch, 2014; Adam et al., 2003; Miller, 1982; Rosenbaum, 1983). In this task, using a four-choice task, a response pre-cue indicates a subset of possible responses in the subsequent trial. In this finger-cueing paradigm, there is usually a clear benefit with hand pre-cues (i.e. left vs. right hand) relative to finger cues, which enable preparation of homologous fingers (i.e. index vs. middle finger) of the two hands (see, e.g., Adam & Koch, 2014, for discussion). Note though that in the explicit finger cueing paradigm, the information conveyed by the explicit pre-cues is statistically identical across the pre-cueing conditions. This is different in the present situation, where incidental learning may occur, so that the predictability of the hand sequence acts as an “implicit” pre-cue. In contrast to an explicit pre-cue, however, the informational value needs to be learnt before it can be used for response preparation. This learning effect seemed to act in addition to general hand-based priming, or response conflict, that occurs during hand repetitions even in random sequences.

In the present incidental learning context, it is notable that participants did not mention the partial predictability very clearly, suggesting that this particular effector sequence learning effect is mostly implicit. Similar observations have also been made in the context of learning other abstract sequences (e.g., Goschke & Bolte, 2012; Koch et al., 2006), but we would like to note that our study was not designed to dissociate implicit learning from explicit learning (see Esser et al., 2022, for a recent review and discussion). Therefore, we might tentatively assume that the present case of effector-sequence learning was not associated with high degree of explicit sequence awareness, leaving it to future studies to examine the more specific contributions of explicit vs. implicit learning processes to the observed learning benefits in performance.

Verwey and Clegg (2005) reported that an effector-dependent representation of a sequence occurs when the sequence is practiced exclusively and extensively with the fingers of one hand. They suggested that this effector-dependent sequence learning is based on mechanisms in which biomechanical aspects constrain the independence of the movements of single effectors (fingers of one hand in this case). For example, responding with one specific finger would have a secondary effect on neighbouring fingers, which could facilitate the creation of effector-dependent representations. A similar mechanism could have resulted in the performance improvements seen in hand repetitions, where a finger response is followed by the neighbouring finger’s response although the order of responses would not be predictable.

Learning to predict either the repetition of a hand or an imminent shift between hands may require some kind of integration of the two contralateral body segments, which may be distinct from those strategies or mechanisms involved in bilateral or cross-limb transfer. In the context of motor skill acquisition, for example, bilateral transfer has been related to the application of cognitive strategies that can be generalized across limbs (Malfait & Ostry, 2004; Yadav & Mutha, 2020). Similar assumptions of abstract representations, such as representations in terms of visuo-spatial and motor coordinates, have been proposed to enable transfer between effectors and effector-independent knowledge when learning action sequences (Panzer et al., 2009; Park & Shea, 2002). For example, these possibilities could be assessed using a limb crossing postural configuration of the effectors.

We believe it unlikely, however, that any generalizable representation of the hand sequence was acquired in the context of the present SRT task. Instead, it is conceivable that incidental acquisition of the hand sequence is analogous to “model-free” habit learning that may resemble a distinct associative learning mechanism, which determines which actions lead to favourable outcome, e.g. in terms of correctly predicted target stimuli, and which may occur in the context of goal-directed associative learning (Daw et al., 2005; Doll et al., 2012). Based on theories of excitatory and inhibitory interhemispheric interactions via the corpus callosum of the human brain that enable both independent and integrated processing of the hemispheres (Bloom & Hynd, 2005; van der Knaap & van der Ham, 2011), we speculate that strong effects of learning hand repetitions compared to hand alternations express initially strong interhemispheric inhibitory influences that subside with practice until excitatory interhemispheric interactions gain traction that facilitate the learning of hand alternations. The borderline sequence learning observed in finger shifts that included hand alternations could mean that the learning period was not of sufficient length.

The observations made in the present study and their interpretation of evidence for incidental hand sequence learning need to be considered tentative in nature as alternative explanations cannot be ruled out at this point. For example, the spatial distance between the individual response locations and a delay resulting from the requirement to subsequently shift the attentional focus from one location to another could have led to shorter latencies for within-hand repetitions involving neighbouring fingers compared to hand alternations. It is necessary in future studies, therefore, to contrast transitions between non-neighbouring finger positions within a hand to transitions between fingers of different hands taking into account the spatial distance between response locations. In addition, three within-hand response alternatives ought to be tested to avoid predictability of finger transitions. It is also conceivable that participants learnt the sequence of possible response locations. Therefore, potential confounds between anatomical relationships of fingers and hands and their locations depending on specific hand postures in peripersonal space need to be resolved, for example by varying hand postures (palms up/down, crossed/uncrossed). Effector sequence learning should occur independent of these factors.

In conclusion, our study demonstrates that in a SRT task, in which the responses were associated with a predictable hand sequence but where the specific finger actions were largely unpredictable, humans were able to improve their performance relative to unpredictable hand sequences. Participants appeared to be more sensitive to hand repetitions, where a succeeding target stimulus and the associated response was theoretically predictable as direct finger repetitions were not possible, compared to shifts between hands, which nevertheless demonstrated a small benefit compared to random hand sequence. Together, our novel findings represent first evidence for learning abstract effector sequences in the absence of specific response sequences.

## Data Availability

Processed data and MATLAB scripts are available for download from the figshare.com database.
